# PCB-153 Shows Different Dynamics of Mobilisation from Differentiated Rat Adipocytes during Lipolysis in Comparison with PCB-28 and PCB-118

**DOI:** 10.1371/journal.pone.0106495

**Published:** 2014-09-11

**Authors:** Caroline Louis, Gilles Tinant, Eric Mignolet, Jean-Pierre Thomé, Cathy Debier

**Affiliations:** 1 Institut des Sciences de la Vie, Université catholique de Louvain, Louvain-la-Neuve, Belgium; 2 Laboratoire d'Ecologie animale et d'Ecotoxicologie, Université de Liège, Liège, Belgium; Wayne State University, United States of America

## Abstract

**Background:**

Polychlorinated biphenyls (PCBs) are persistent organic pollutants. Due to their lipophilic character, they are preferentially stored within the adipose tissue. During the mobilisation of lipids, PCBs might be released from adipocytes into the bloodstream. However, the mechanisms associated with the release of PCBs have been poorly studied. Several *in vivo* studies followed their dynamics of release but the complexity of the *in vivo* situation, which is characterised by a large range of pollutants, does not allow understanding precisely the behaviour of individual congeners. The present *in vitro* experiment studied the impact of (*i*) the number and position of chlorine atoms of PCBs on their release from adipocytes and (*ii*) the presence of other PCB congeners on the mobilisation rate of such molecules.

**Methodology/Principal Findings:**

Differentiated rat adipocytes were used to compare the behaviour of PCB-28, -118 and -153. Cells were contaminated with the three congeners, alone or in cocktail, and a lipolysis was then induced with isoproterenol during 12 hours. Our data indicate that the three congeners were efficiently released from adipocytes and accumulated in the medium during the lipolysis. Interestingly, for a same level of cell lipids, PCB-153, a hexa-CB with two chlorine atoms in *ortho*-position, was mobilised slower than PCB-28, a tri-CB, and PCB-118, a penta-CB, which are both characterised by one chlorine atom in *ortho*-position. It suggests an impact of the chemical properties of pollutants on their mobilisation during periods of negative energy balance. Moreover, the mobilisation of PCB congeners, taken individually, did not seem to be influenced by the presence of other congeners within adipocytes.

**Conclusion/Significance:**

These results not only highlight the obvious mobilisation of PCBs from adipocytes during lipolysis, in parallel to lipids, but also demonstrate that the structure of congeners defines their rate of release from adipocytes.

## Introduction

Polychlorinated biphenyls (PCBs) are a class of environmentally persistent pollutants that biomagnify throughout food chains. Ingestion of contaminated food, and especially fat-rich animal products, represents 90% of the mean uptake of humans [1]. Adipose tissue is then the main reservoir for the storage of these highly lipophilic molecules [2]. The cytoplasm of adipocytes is almost exclusively composed of lipid droplets (LDs) [3], which appear to be the principal targets for PCBs [4]. These cells therefore have an enormous capacity to accumulate lipophilic pollutants [5].

During periods of weight loss in humans, lipids from adipose tissue are mobilised, leading to an increase of PCB concentrations in this tissue [6], [7]. Evidence from wildlife indicates same trends [8]–[14]. This phenomenon suggests that PCBs are less efficiently mobilised from adipocytes than fatty acids. However, a release of PCBs in the blood circulation does occur during such periods of weight loss and appears to become more important when the adipose stores are already significantly reduced [2], [6], [15]–[18]. In addition to being a reservoir, the adipose tissue can thus also be an internal source of lipophilic pollutants for the rest of the body [2]. Once in the bloodstream, pollutants are able to contaminate other tissues or be transferred in maternal milk. The exposure to PCBs is associated to adverse effects on human and animal health [19], [20]. Among others, PCBs are involved in endocrine disruption, immuno- and neuro-toxicity as well as in the development of cardiovascular diseases and type-2 diabetes [21]–[25]. A correlation between the rise of persistent organic pollutants (POPs), such as PCBs, in the serum and the alterations of skeletal muscle oxidative capacity has been suggested in humans [16]. Furthermore, individuals who underwent bariatric surgery exhibited a positive association between POP serum levels and a diminished improvement of lipid values and liver markers [6].

Even if several *in vivo* studies report a release of PCBs from adipose tissue during lipolytic process [6], [10], [13]–[15], little is known concerning the chemical and biochemical factors that govern their mobilisation and transfer into the circulation. *In vivo* studies on long-term fasting wild animals report an impact of the fasting stage as well as the degree of lipophilicity of PCB congeners on their dynamics of mobilisation from the adipose tissue [8], [10], [13], [14]. The transfer from adipose tissue into the blood circulation appears to be selective and strongly dependent on the log K_ow_ value of the compounds, with less lipophilic PCBs being more efficiently released. *In vivo* models being usually complex, the *in vitro* cultures of adipocytes would be useful to precisely understand the mobilisation of PCB congeners as a function of their chemical structure. A recent study from our group investigated the dynamics of accumulation of three PCB congeners, differing in the position and number of their chlorine atoms (PCB-28, log K_ow_ = 5.71; PCB-118, log K_ow_ = 6.57 and PCB-153, log K_ow_ = 6.80) in cultured adipocytes [26]. The accumulation profile revealed significant differences between PCB congeners. Their release during lipolysis was however not investigated.

In this study, we followed and compared the dynamics of mobilisation of PCB-28, PCB-118 and PCB-153 from *in vitro* differentiated rat adipocytes. Cells were contaminated by the three congeners, added individually or in cocktail, at the same concentrations in the culture medium. Lipolysis was then triggered over 12 hours with a lipolytic medium supplemented with isoproterenol, a well-known synthetic catecholamine [27], [28]. The levels of PCBs in the extracellular medium and adipocytes were regularly assessed. The present experiment allowed (*i*) to estimate the impact of the number and position of chlorine atoms of PCBs on their release from adipocytes and (*ii*) to assess the impact of the presence of other PCB congeners on the mobilisation dynamics of such molecules.

## Experimental Procedures

### Primary cultures of rat adipocytes

Differentiated rat adipocytes were obtained and cultured as described previously [5], [28]. Experimental procedures in animals were approved by the Animal Care and Use Committee of the Université catholique de Louvain (#103201) and were performed in accordance with the “Principles of Laboratory Animal Care” (NIH Publication 85–23). Two-month-old male Wistar rats (Centre d'Elevage Janvier, Le Genest Saint Isle, France) were sacrificed by decapitation. The fat tissue of the stromal-vascular fraction was sampled and then digested in a solution of collagenase (1250 U/ml type II; Sigma-Aldrich, Bornem, Belgium). The digested tissue underwent three filtrations and three centrifugations in order to obtain a final pellet of stromal-vascular cells, which was suspended in a medium composed of Dulbecco's Modified Eagle Medium (DMEM, 4.5 g/l glucose, Gibco-Invitrogen, Merelbeke, Belgium), 10% (*v:v*) heat-inactivated foetal bovine serum (FBS, PAA, A&E Scientific, Marcq, Belgium) and an antibiotic and antifungal mixture. Cells were seeded at a mean density of 18,000 cells per cm^2^ on 6-well plates (Corning CellBIND Surface, Corning, Elscolab, Kruibeke, Belgium) (day 0) and incubated at 37°C in a humidified atmosphere containing 10% CO_2_ in air for 24 hours to allow cell sedimentation and adhesion. Twenty-four hours after the isolation of progenitor cells (day 1), the medium was replaced by a differentiation medium composed of DMEM (4.5 g/l glucose), 10% (*v:v*) heat-inactivated FBS, 100 U/ml penicillin – 100 U/ml streptomycin – 250 ng/ml amphotericin B mixture (Lonza, Verviers, Belgium), 10 nM dexamethasone (Sigma-Aldrich), 10 µM ciglitizone (Sigma-Aldrich) and 5 µg/ml insulin (Sigma-Aldrich). This medium was renewed every 48 hours until day 10 in order to obtain differentiated adipocytes.

### Cell treatment

At day 10, adipocytes were incubated with a medium supplemented with PCB congeners (Dr. Ehrenstorfer GmbH, Ausburg, DE) during 12 hours (37°C – 10% CO_2_ in air). The PCBs were added to the culture medium as ethanolic solution and four conditions of PCB contamination were tested: (*i*) 2,4,4′-trichlorobiphenyl (PCB-28); (*ii*) 2,3′,4,4′,5-pentachlorobiphenyl (PCB-118); (*iii*) 2,2′,4,4′,5,5′-hexachlorobiphenyl (PCB-153); (*iv*) an equimolar mixture of three PCB congeners (PCB-28, -118 and -153), also called cocktail of PCBs. In all conditions of contamination, each PCB was added to the culture medium at a concentration of 300 nM, which is within the range of concentrations found in *in vivo* and *in vitro* studies [4], [29], [30]. Impact of the ethanol vehicle was tested earlier [4].

### Lipolysis experiment

At day 11, lipolytic process was induced to differentiated adipocytes as previously described in Louis et al. [28]. The differentiation medium in contact with adipocytes was removed and replaced by a lipolytic medium composed of DMEM (1.0 g/l glucose, Gibco–Invitrogen), 5% (*v:v*) heat-inactivated FBS, 2% (*w:v*) bovine albumin (Sigma-Aldrich) and 1 µM isoproterenol (Sigma-Aldrich). The lipolytic medium was renewed every 3 hours and the process was carried out for 12 hours. In the same way as in Louis et al. [28], cells from one plate (*i.e.* cells coming from the same PCB contamination) were collected every 3 hours and pooled in order to assess the cellular PCB and protein contents as well as the levels of fatty acids of cellular neutral lipids (NLs). Likewise, free fatty acids (FFAs), glycerol and PCBs released in the extracellular medium were quantified every 3 hours in all conditions.

### Cellular protein assessment

Every 3 hours, cells were washed with phosphate-buffer saline (Sigma-Aldrich) at 37°C and then collected in an aqueous solution composed of 35 mM sodium dodecyl sulfate (Merck, Darmstadt, Germany), 60 mM Tris buffer (Merck) and 10 mM ethylenediaminetetraacetic acid (Sigma-Aldrich). After homogenisation, the cellular protein content was quantified by using the Bicinchoninic Acid Protein Assay kit (Sigma-Aldrich) with bovine serum albumin (Sigma-Aldrich) as calibration curve [28].

### Cellular neutral lipid assessment

Cells were collected as described for the determination of protein content. The method used for the extraction and the isolation of the NL fraction (i.e. triglycerides (TGs), diglycerides, monoglycerides and cholesterol esters) from cell lysates is described in details in Louis et al. [28]. Briefly, the lipids were extracted with a mixture of chloroform/methanol/water (2∶2∶1, *v:v:v*) (Biosolve, Valkenswaard, The Netherlands) containing triheptadecanoin (Larodan, Malmö, Sweden) used as internal standard. After centrifugation, the supernatant was discarded; the chloroform phase was evaporated and samples were then suspended into 200 µl chloroform. In order to isolate NLs, samples were loaded on solid-phase extraction columns (Bond Elut NH_2_, 200 mg, Varian, Middelburg, The Netherlands). The NL fraction collected with chloroform/2-propanol (2∶1, *v:v*) (Biosolve) was evaporated and a methylation step was performed by adding 0.1 M KOH (Sigma-Aldrich) in methanol at 70°C for 1 hour and then, by adding 1.2 M HCl in methanol at 70°C for 15 min. The addition of hexane followed by deionized water allowed extracting the fatty acid methyl esters by a centrifugation step. Those were separated by gas chromatography [31]. Each peak was then identified and quantified by comparison with pure methyl ester standards (Larodan and Nu-Check Prep, Elysian, MN, USA). Data were processed with the ChromQuest 4.2 software (ThermoFinnigan, Milan, Italy). Thereafter, results were expressed by moles of fatty acids in cellular NLs. For the sake of simplicity, we refer to µmol NLs/mg protein in the text [28].

### Extracellular free fatty acid assessment

FFA contents in the lipolytic medium were quantified with the Wako NEFA HR kit (Sopachem, Eke, Belgium) following the manufacturer's instructions [28].

### Extracellular glycerol assessment

Glycerol released in the extracellular medium was measured with an *in vitro* enzymatic colorimetric test using glycerol-3-phosphate-oxidase (Diasys Free Glycerol FS kit, Sopachem) according to the manufacturer's instructions.

### PCB assessment

At each studied time of the lipolysis, cells and extracellular medium were collected in EPA vials (Alltech, Lokeren, BE) with 5 ml of *n*-hexane (Biosolve) in order to perform a liquid-liquid extraction by a 10-min shaking. The hexane phase was transferred into a tube and PCB-112 (Dr. Ehrenstorfer GmbH) was added as internal standard. All samples were then purified by acid and Florisil clean-up steps as described in Debier et al. [8]. Purified samples were collected in *n*-hexane. Five µl of anhydrous nonane (Sigma-Aldrich) were added to samples and solvent was then evaporated under a gentle stream of nitrogen. The purified extracts were suspended into a hexane solution of Mirex (200 pg/µl) (Dr. Ehrenstorfer GmbH) used as internal standard for the correction of the extract volume injected in GC/MS. PCB congeners were separated and quantified with a gas chromatograph (GC Trace, ThermoFinnigan) equipped with an automatic split/splitless type injector (CTC Analytics, Zwingen, Switzerland), a fused silica capillary column (30 m × 0.25 mm internal diameter; 0.25 µm film) (Rxi-5ms, Restek, Bellefonte, PA, USA) and a mass spectrometer (Trace DSQ, ThermoFinnigan). The system used helium as carrier gas at a constant flow rate of 1.1 ml per minute. The temperature of injector was 230°C. The oven temperature program was as follows: 2 min at 60°C, gradual heating from 60 to 140°C at the rate of 20°C per minute, 1 minute at 140°C, gradual heating from 140 to 290°C at the rate of 2.5°C per minute, 10 minutes at 290°C and gradual cooling from 290°C to 60°C at the rate of 10°C per minute. Molecules were sent to mass spectrometer by the line transfer at 290°C. The ion source of the detector was kept at 230°C. PCBs were identified according to their retention time. Data were recorded using XCalibur 1.3 software (ThermoFinnigan). Quantification was performed by comparison to an external standard composed of 28 congeners (IUPAC numbers: PCB-8, -18, -28, -44, -52, -66, -77, -81, -101, -105, -114, -118, -123, -126, -128, -138, -153, -156, -157, -167, -169, -170, -180, -187, -189, -195, -206 and -209) in a certified calibration mixture (AccuStandard, New Haven, CT, USA). Five dilutions (concentration ranging from 25 to 500 pg/µl) were used in order to draw a linear calibration curve for each PCB.

### Quality control

Blanks were run with sample series to control extraction and clean-up steps. The PCB recovery was calculated on the basis of the internal standard, PCB-112. Results were accepted only if the recoveries were between 70 and 130% according to EC [32]. All results were corrected to obtain 100% recovery [8]. The quality control was assessed through an interlaboratory comparison.

### Cytotoxicity assessment

The potential cytotoxicities of the PCBs and the lipolytic treatment were assessed by measuring the release of lactate dehydrogenase (LDH) in the extracellular medium. The activity of LDH was determined using the cytotoxicity detection kit (Roche Diagnostics, Vilvoorde, Belgium) according to the manufacturer's instructions in (*i*) the differentiation medium after 12 hours of PCB exposure and (*ii*) the lipolytic media collected every 3 hours during the lipolytic process. Before the PCB exposure and the lipolysis, some cells were lysed with 1% Triton x-100 (Sigma-Aldrich) and were used as full toxicity control [28]. No treatments appeared toxic (< 5% of control) as compared to the full toxicity control (results not shown).

### Statistical analysis

Data are presented as means ± SEM of three independent experiments for the conditions with PCB congeners alone and means ± SEM of five independent experiments for the condition with the cocktail of PCBs. The statistical analysis was performed by SAS 9.3 software (SAS Institute Inc., Cary, USA). Differences between treatments were assessed with mixed linear models and a Tukey's test [28]. Differences were deemed significant at *p*-values<0.05.

## Results

### 1. Incorporation of PCBs in adipocytes

At day 10, differentiated rat adipocytes were exposed to four different treatments of PCBs during a 12-hour period. Three PCB congeners (PCB-28, -118 and -153) were added to the culture medium at a concentration of 300 nM, alone or in cocktail. Assessment of PCBs within adipocytes was carried out before the induction of lipolysis. The concentration of each congener in adipocytes, expressed as nmol per unit of cellular protein, was statistically similar, whatever the kind of contamination (alone or in cocktail) (0.328<*p*<1.000) (Table 1). Same conclusions were drawn when the results were expressed per unit of NLs (0.207<*p*<1.000). Since the dynamics of accumulation of PCBs in cells vary with cellular lipid content [26], the levels of cellular NLs were quantified and no statistical difference was noted (0.457<*p*<0.978) (Table 1).

**Table 1:** Efficient accumulation of PCBs in adipocytes during a 12-hour period.

	Contamination by a PCB congener alone:			Contamination by a cocktail of three PCB congeners:		
	PCB-28	PCB-118	PCB-153	PCB-28	PCB-118	PCB-153
PCBs (nmol/mg protein)	1.7±0.1	2.0±0.1	1.8±0.1	1.8±0.1	2.1±0.1	1.8±0.1
PCBs (nmol/mg NLs)	0.47±0.08	0.62±0.09	0.48±0.08	0.61±0.08	0.70±0.08	0.60±0.08
NLs (µmol/mg protein)	13.6±1.1	13.1±1.3	15.6±1.1	11.4±1.1	11.4±1.1	11.4±1.1

Quantities of PCBs accumulated in differentiated rat adipocytes, expressed in nmol per unit of cellular protein and per unit of neutral lipids (NLs), after a dose of PCBs was added during 12 hours in the culture medium. Quantities of NLs, expressed in µmol per unit of cellular protein are also presented. Data represent the means of (*i*) three independent experiments ± SEM for conditions with one PCB congener alone, (*ii*) five independent experiments ± SEM for conditions with the cocktail of PCBs. There was no significant difference of PCB and NL concentrations between the three PCB congeners, whatever the kind of contamination, alone or in cocktail (*p*>0.05).

### 2. The time-course of lipolysis

**Figure 1:**
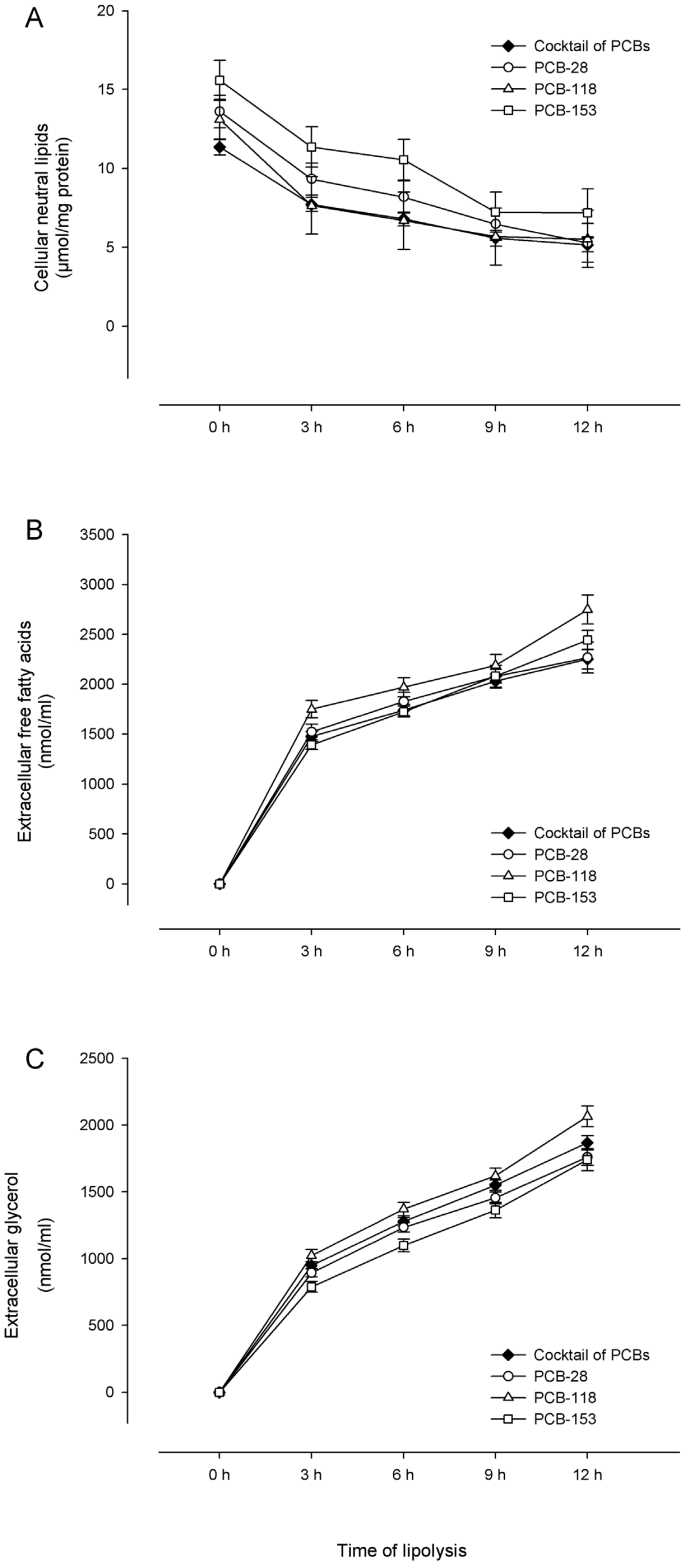
Lipolytic treatment decreased cellular neutral lipids and increased extracellular fatty acids and glycerol. At day 11, differentiated rat adipocytes, which were previously contaminated with PCBs, were incubated with a lipolytic medium supplemented with 1 µM isoproterenol. We renewed the lipolytic medium every 3 hours for 12 hours. Cellular neutral lipids (corresponding to µmol of fatty acids in cellular neutral lipids) were expressed per mg of total cell protein (A). A significant decrease of cellular lipid contents was noted throughout the lipolytic process for the conditions with PCB-28 (*p* = 0.005) and the cocktail of PCBs (*p* = 0.029). The decrease was slighter for the conditions with PCB-118 (*p* = 0.298) and PCB-153 (*p* = 0.097). Total extracellular free fatty acids (B) and total extracellular glycerol (C) were expressed per ml of medium. Quantities of total free fatty acids and glycerol in the medium were obtained by adding the quantities released during the periods of 3 hours (e.g. total free fatty acids at 6 hours correspond to the sum of total free fatty acids released between 0 and 3 hours and between 3 and 6 hours). A significant increase of total free FAs and total glycerol was observed over the 12-hour lipolytic treatment (*p*<0.001 for all conditions). Data represent the means of (*i*) three independent experiments ± SEM for conditions with one PCB congener alone, (*ii*) five independent experiments ± SEM for conditions with the cocktail of PCBs.

At day 11, adipocytes contaminated with PCBs (alone or in cocktail) were incubated with a lipolytic medium supplemented with 1 µM isoproterenol, which was replaced every 3 hours for 12 hours. Since the aim of this work was to study the kinetics of PCB release during the mobilisation of lipids in adipocytes, we firstly ensured of the efficiency of lipolysis. Cellular NLs as well as FFAs and glycerol released in the culture medium were quantified over 12 hours (Figure 1). The FFA and glycerol initially present in the lipolytic medium, before being in contact with adipocytes, were subtracted from each result, leading to a value of 0 at 0 hour (Figure 1B–C). The total FFAs and glycerol released by the adipocytes throughout a given period were calculated by adding the quantities measured at each period of 3 hours. For example, the amount of total FFAs or glycerol released after 6 hours of lipolysis corresponds to the sum of FFAs or glycerol released between 0 and 3 hours and between 3 and 6 hours [28].

A significant decrease of cellular NLs was observed between early and late lipolysis for the conditions with the PCB-28 (*p* = 0.005) and cocktail of PCBs (*p* = 0.029). The cell NLs slightly decreased over 12 hours within adipocytes contaminated with PCB-118 (*p* = 0.298) and PCB-153 (*p* = 0.097). For all conditions, the greatest reduction occurred during the first 3 hours of the experiment (Figure 1A). Indeed, a mean loss of 57±3% initial NLs was noted between 0 and 12 hours, whereas after 3 hours of lipolysis, adipocytes already lost 33±6% of NLs on average, compared to the initial levels (0.372<*p*<0.721 for all conditions of contamination between 0 and 3 hours). The comparison of NL levels between the four PCB treatments at a given period did not highlight any difference (0.255<*p*<1.000).

The decrease of cellular lipid contents was accompanied by a significant increase of total FFAs in the lipolytic medium over the 12-hour period (*p*<0.001) (Figure 1B). The increase of total FFAs was more pronounced during the first 3 hours of lipolysis, during which an average release of 63±4% of total FFAs occurred (*p*<0.001). FFA concentrations then continued to increase slightly, but not significantly, between the subsequent consecutive periods (0.491<*p*<0.985 for all conditions of contamination between 3 and 6 hours, 6 and 9 hours, 9 and 12 hours). No difference in the release of FFAs was noted between the four conditions of contamination at a given time of the lipolytic process (0.273<*p*<1.000). As for total FFAs, contaminated adipocytes released a significant amount of total glycerol throughout the lipolytic process (*p*<0.001 between 0 and 12 hours) (Figure 1C). The major part of the release (49±3%) also occurred during the first 3 hours of lipolysis for all conditions (*p*<0.001) (Figure 1C). Here again, no difference in the release of glycerol was noted between the four conditions of PCB contamination over the 12-hour period (0.119<*p*<0.999). Taking all conditions of PCB contamination together, the FFA/glycerol ratios lied between 1.4 and 1.8.

### 3. Comparative dynamics of mobilisation of PCB-28, -118 and -153 present in cocktail in adipocytes

In the present section, we compared the dynamics of mobilisation of PCB-28, -118 and -153 (present in cocktail within the cells) from the same adipocytes undergoing a lipolytic process over a 12-hour period. In order to strictly compare the different mobilisations between congeners, we expressed the results as percentages of the amounts initially present in adipocytes (i.e. at 0 hour). The potential presence of PCBs in the lipolytic medium, before being in contact with the cells, has been tested and was negligible (result not shown). The proportions of PCBs in the lipolytic medium at 0 hour were thus set at 0%. Regarding the subsequent studied times of the lipolysis (3, 6, 9 and 12 hours), the PCB levels in the medium were calculated by adding the quantities evaluated at each period of 3 hours. For example, the amounts of each PCB congener released after 6 hours of lipolytic treatment correspond to the sum of PCB released between 0 and 3 hours and between 3 and 6 hours.

**Figure 2:**
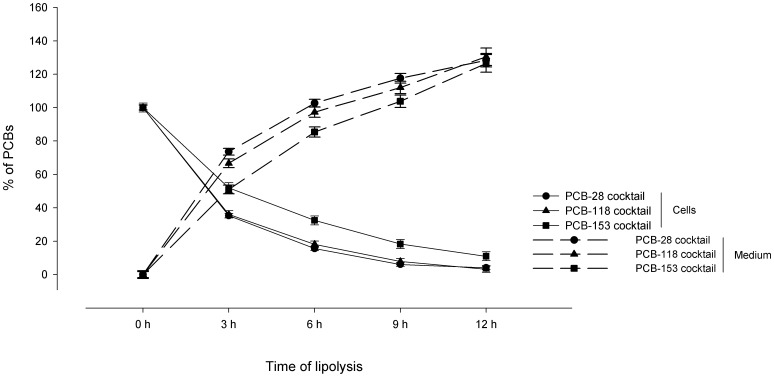
Lipolytic treatment decontaminated the adipocytes, inducing an accumulation of PCB congeners in the extracellular medium. At day 11, contaminated adipocytes underwent a lipolytic process. The PCB contents as well as the proportions of PCBs released in the extracellular medium were assessed every 3 hours. For each PCB congener, all results were expressed in percentage of the amounts initially present in the cells. Proportions of one PCB congener in the medium were obtained by adding the amounts released during periods of 3 hours (e.g. proportion of one congener at 6 hours corresponds to the sum of this congener released between 0 and 3 hours and between 3 and 6 hours). An important drop of cell PCB proportions was observed during the first 6 hours of lipolysis (*p*<0.001) and was followed by a more moderate decrease of the cell PCB percentages during the last 6 hours (*p*<0.001). In parallel, an important increase of PCBs was noted in the extracellular medium during the first half of lipolysis (*p*<0.001) and was followed by a slower accumulation during the second half of lipolysis (0.001<*p*<0.006). Data represent the means of five independent experiments ± SEM.

In parallel to the lipid mobilisation, there was a release of PCBs from adipocytes into the culture medium (Figure 2). An important drop of the PCB cellular content occurred during the first half of the lipolytic process (*p*<0.001 between 0 and 6 hours for the three PCBs). It was followed by a slight, but still significant reduction of PCB-28, -118 and -153 in adipocytes during the second part of the lipolysis (*p*<0.001 between 6 and 12 hours for the three PCBs). Accordingly, a reversed trend was noted in the lipolytic medium: the proportions of PCB-28, -118 and -153 accrued during the first 6 hours (*p*<0.001 between 0 and 6 hours for the three PCBs). The PCB accumulation in the culture medium was more moderate, but still significant (0.001<*p*<0.006), during the second part of the lipolytic process.

Even if all congeners dropped in the cells and increased in the culture medium, the dynamics of release of PCB-153 somewhat differed from those of PCB-28 and -118. Indeed, despite the fact that the amounts of the three congeners within adipocytes were statistically similar before the beginning of the lipolysis (Table 1), the percentages of PCB-153 in adipocytes remained higher than the ones of PCB-28 and PCB-118 at 3, 6 and 9 hours of lipolysis (0.001<*p*<0.032 for PCB-28; 0.031<*p*<0.037 for PCB-118). The difference disappeared at 12 hours between PCB-153 and PCB-28 (*p* = 0.139), but remained significant between PCB-153 and PCB-118 (*p* = 0.027). On the other hand, the cellular percentage of PCB-28 and PCB-118 did not differ between each other throughout the lipolytic process (0.373<*p*<1.000). The slower mobilisation of PCB-153 from adipocytes was reflected by a slower increase in the culture medium as compared to PCB-28 and PCB-118 after 3 hours of lipolysis (*p*<0.001 for the both comparisons). After 6 hours, the accumulation in the medium was weaker for PCB-153 than PCB-28 (*p* = 0.027) and similar between PCB-153 and PCB-118 (*p* = 0.178). The proportions of PCB-153 were then similar to those of PCB-28 and -118 for the subsequent hours (0.275<*p*<0.986 at 9 and 12 hours). On the other hand, the percentages of PCB-28 and -118 in the extracellular medium were similar to each other over the lipolysis (0.429<*p*<1.000).

### 4. Impact of the presence of other PCB congeners on the dynamics of mobilisation of PCB-28, -118 and -153

We investigated if the dynamics of mobilisation of one PCB congener was influenced by the presence of the other congeners. To achieve this goal, we contaminated adipocytes either with one of the three PCB congeners (PCB-28, -118 or -153) or with a cocktail of the three congeners. Here also, the results are expressed as percentages of the amounts initially present in adipocytes. As previously described, the proportions of PCBs just before the lipolytic process (i.e. 0 hour) was set to 0% since no PCB congener was quantified in the initial lipolytic medium. Here also, the total PCB congeners released by the adipocytes throughout a given period were calculated by adding the quantities measured at each period of 3 hours.

**Figure 3:**
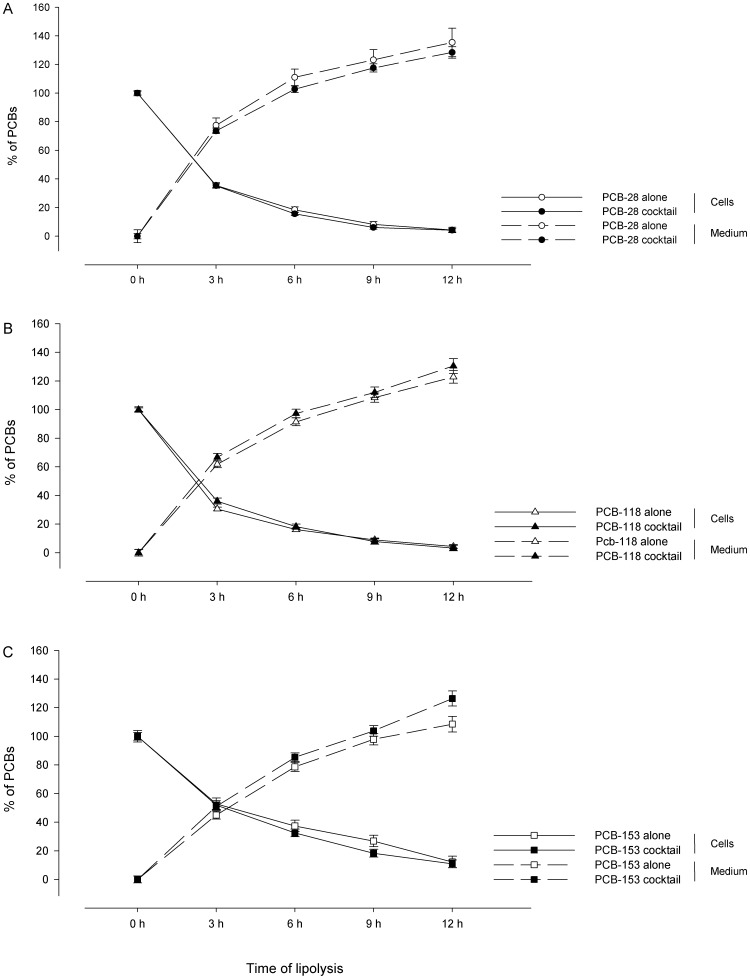
Presence of other congeners did not influence the dynamic of PCB mobilisation. At day 11, differentiated rat adipocytes, which were previously contaminated with either individual PCB congeners or with a cocktail of PCBs, underwent a lipolytic process. The cellular levels of PCBs before the lipolytic process were quantified and set at 100%. During 12-hour period of lipolysis, the contents of PCB congeners within adipocytes and in the extracellular medium were assessed every 3 hours. The results for PCB-28 (A), PCB-118 (B) and PCB-153 (C) were expressed by the percentage of initial amounts of each congener. Within a condition of contamination, proportions of one PCB congener in the medium were obtained by adding the quantities released during the periods of 3 hours (e.g. proportion of one congener at 6 hours corresponds to the sum of this congener released between 0 and 3 hours and between 3 and 6 hours). At each given time of lipolytic treatment, no differences were noted between the proportions of each PCB (i.e. PCB-28, -118 and -153) in both conditions of contamination (i.e. congeners alone or in cocktail), either in the cells or in the lipolytic medium (0.122<*p*<0.916). Only the percentage of PCB-153 in the lipolytic medium was lower when taken alone as compared to the condition in cocktail after 3 hours of lipolysis (*p* = 0.046). Data represent the means of (*i*) three independent experiments ± SEM for conditions with one PCB congener alone, (*ii*) five independent experiments ± SEM for conditions with the cocktail of PCBs.

At each studied time throughout the 12-hour period, similar proportions of PCB-28 (Figure 3A), PCB-118 (Figure 3B) and PCB-153 (Figure 3C) within adipocytes were quantified between both conditions of contamination (i.e. congeners alone and in cocktail) (0.134<*p*<0.934). Accordingly, no difference was noted between the percentages of PCBs released in the lipolytic medium in contact with adipocytes in both conditions of contamination (0.122<*p*<0.916), except after 3 hours of lipolysis, where the proportion of PCB-153 measured in the medium were higher when it was added in cocktail than when it was added alone (*p* = 0.046).

## Discussion

### Considerable accumulation of PCBs within adipocytes

Differentiated rat adipocytes were exposed to three targeted PCB congeners (PCB-28, -118 and -153), which differ by the number and the position of the chlorine atoms [19], [33]. After 12 hours, similar concentrations of PCBs were stored within adipocytes. Same observations were already drawn after 4 hours of exposure for the same congeners [26]. The different molecular structures of PCBs did thus not seem to influence their accretion within adipocytes on long term. Previous studies from our group highlighted the importance of the amount of cellular NLs, acting as a trap for the accumulation of PCBs within adipocytes [5], [26]. The fact that cellular NL levels were similar between our experimental conditions is most probably at the origin of the identical accumulation of PCBs within adipocytes. While PCB concentrations in the culture medium were in the same range than those measured in the human serum [29], [30], the PCB levels found in cultured adipocytes after 12 hours of incubation (data from Table 1 are equivalent ∼175 ng PCB congeners per mg NLs) were much higher than those measured *in vivo*, in the human adipose tissue (from 0.02 to 0.66 ng total PCBs per mg lipids) [34]–[38]. It reflects a high propensity of differentiated rat adipocytes to store PCBs [4]. Such differences have already been noticed previously and the reasons are discussed in details elsewhere [5]. Briefly, the higher *in vitro* concentrations of PCBs within adipocytes could result from the extended contact between the cells and the contaminated culture medium (12 hours). On the contrary, in the *in vivo* situation, the PCB congeners, transported in the circulation by lipoproteins and plasma albumin [33], [39], [40], are in continual movement thanks to the blood flow. In addition, the culture medium contains only a low concentration of serum (10%) [4], and therefore very low levels of lipoproteins and albumin, which could contribute to a smaller retention of PCBs in this hydrophilic compartment and a higher storage in the lipophilic compartment represented by adipocytes. The differentiated rat adipocytes are also organised as a monolayer whereas *in vivo* adipose tissue shows a complex 3D-structure. Furthermore, lipolysis, which occurs regularly *in vivo*, may lead to the mobilisation of PCBs from adipocytes. Finally, the circulating PCBs may be taken up by other tissues such as the liver.

### Similar dynamics of mobilisation between cellular lipids and PCBs

Once the lipolytic pathway was induced, adipocytes started to mobilise their lipid content. A decrease of the cellular NLs could be observed throughout the 12-hour experiment, with a more pronounced lipolytic action during the first 3 hours. This sharper decrease of NL content at early lipolysis was in accordance with our previous study [28]. As a result of the mobilisation of cellular NLs, FFAs and glycerol were released in the extracellular medium. The lipolytic treatment also led to the release of PCBs from adipocytes to the extracellular medium. The dynamics of mobilisation of PCBs exhibited some parallelism with those of cellular lipids, as the major part of PCB discharge occurred during the first hours of lipolysis as well. Previously, it was shown that *in vitro* epididymal adipocytes isolated from rats also unloaded PCB-153 during a lipolytic treatment of 50 min with 0.8 µM isoproterenol [41]. The release of PCBs might accompany the mobilisation of cell lipids, which agrees with previous studies on the behaviour of dioxins [42], [43]. In addition, cellular TG content is an important parameter governing the accumulation of PCBs in adipocytes [5], [26]. PCBs are stored almost exclusively within the LDs [4]. As this lipophilic pool is reduced during lipolysis [28], the capacity of storage is thus also lessened, promoting the release of PCBs in the extracellular medium, where they could be tightly associated with diverse lipoproteins (present in the 5% serum) and bovine albumin (2%).

Although an obvious release of PCBs occurred from adipocytes during the lipolytic experiment, some molecules of PCBs could be taken up again by the cells as previously suggested in *in vivo* studies [13], [14]. This phenomenon is well known for FFAs, which are partly reabsorbed by adipocytes and re-esterified into newly synthesized TGs [28], [44]. A complete hydrolysis of one mole of TGs leads to the release of three moles of free FAs and one mole of glycerol. This could be translated by a free FA/glycerol ratio equivalent to 3.0. However, free FA/glycerol ratios were lower than 3.0 in our experiments, which likely reflects a reuptake of free FAs by the cells. Nevertheless, this phenomenon might have been somewhat limited in our experimental conditions, because of the renewal of the lipolytic medium every 3 hours [28].

Studies investigating the release of POPs from adipose tissue during periods of weight loss in animals and humans usually report an increase of the concentrations of PCBs and related compounds in adipose tissue, despite their significant discharge in the blood circulation [6], [14], [15], [18]. This increase suggests a less efficient mobilisation of PCBs from this tissue than lipids and a concentration of these lipophilic pollutants in the remaining amount of fat cells. The adipose tissue is a macroscopic structure that is irrigated by blood vessels. During adipose tissue lipolysis, it is possible that PCBs are transferred to adipocytes that still contain significant amounts of lipids in their LDs instead of being all released into the circulation. It is also possible that PCBs are released in the bloodstream together with the lipids and then reabsorbed by the adipose tissue as a result of their higher affinity for the remaining lipids present in the cells [13]. Our *in vitro* model differs from the *in vivo* situation among others by the fact that it is characterised by only one layer of cells, which is in direct contact with the extracellular medium that is regularly renewed. A reuptake of PCBs by the cells and/or a migration of PCBs to deeper adipocyte layers that are still filled with fat are thus not possible. Moreover, the high PCB concentrations, which were found in the cultivated adipocytes before the lipolytic induction, might also promote the massive release of congeners in the extracellular medium.

### Differences of release according to the kind of PCB congener

When the three congeners were added in cocktail to the culture medium, we could observe that PCB-153 was less efficiently mobilised from adipocytes than PCB-28 and PCB-118 during the first part of the lipolytic process. This difference however disappeared at 12 hours of lipolysis. The slower mobilisation of PCB-153 from adipocytes reflects the fact that, besides the cellular lipid content, the rate of release is also governed by the physico-chemical properties of the congeners, which are defined by the number and the position of chlorine atoms on the biphenyl core [2]. If we consider the electrostatic potentials of PCBs [26], PCB-153 exhibits a large electron-deficient zone. This characteristic makes this congener rather lipophilic, which is reflected by the higher partition coefficient *n*-octanol/water (log K_ow_ = 6.80). On the other hand, PCB-28 and PCB-118 have a reduced electron-deficient zone, translated by lower log K_ow_ (PCB-28: log K_ow_ = 5.71 and PCB-118: log K_ow_ = 6.57). PCB-153 could thus be more trapped within LDs than PCB-28 and PCB-118 and as a consequence, be released more slowly.

In addition, it was previously observed that a small proportion of PCB-153 was sequestrated in the cell membranes when isolated primary adipocytes absorbed the PCB congeners present in the culture medium for 2 hours [4]. It was not the case for PCB-28 and -118. Likewise, several studies showed that PCB-52 and -153, two di-*ortho*-substituted PCBs, intercalate between membrane phospholipids similarly to cholesterol and have an impact on the membrane fluidity in fish, rodent and chicken cells [45]–[49]. In the present study, the release of PCB-153 from adipocytes could thus be slowed down by its association with cell membranes, as compared to PCB-28 and PCB-118, two mono-*ortho*-substituted congeners. The fact that PCB-153 has two chlorine atoms in the *ortho* position on the biphenyl core induces a more perpendicular layout of the phenyl rings. It means that PCB-153 occupies a larger bulk than PCB-28 and PCB-118, which could be involved in the sequestration of PCB-153 within membranes and its slower mobilisation from adipocytes.

Previous findings from our group, investigating the uptake of PCBs by differentiated adipocytes, highlighted that PCB-28 enters the cells more rapidly than PCB-118 and PCB-153 [4]. If, during lipolysis, a reuptake of PCBs by the cells occurs, PCB-28 might thus be taken up more rapidly than the other congeners, which could lead to an underestimation of the differences of mobilisation kinetics during the lipolytic treatment.

### Similar rate of release when PCBs are present alone or in cocktail

In the *in vivo* situation, tissues are exposed to a cocktail of contaminants that might interact with each other's, regarding either the toxicokinetics or the toxicodynamics of the molecules. Here, we investigated the effect of a simple combination of PCBs (three congeners) on their release by adipocytes during lipolysis. To do this, the discharge of PCB-28, -118 and -153 from adipocytes was followed either alone, or in cocktail (i.e. with two other congeners). In the two conditions of contamination, the dynamics of PCB release were similar, meaning that the mobilisation of PCB congeners was not influenced by the presence of other congeners within adipocytes in these experimental conditions. As noted above, PCB-153 influences the properties of cell membranes. One could thus have expected that this congener could influence the dynamics of release of other PCBs from the cells.

## Conclusion

Our results showed an efficient accumulation of PCB-28, -118 and -153 in adipocytes. Once lipolysis was induced, the congeners were massively mobilised from cells into the culture medium, in parallel with the release of lipids. The dynamics of discharge however differed between the three investigated congeners. The release of PCB-153 was slightly but significantly slower than the ones of PCB-28 and -118. The phenomenon might be explained by the fact that PCB-153 is more lipophilic than the two other congeners and could thus be more trapped in LDs. In addition, PCB-153 being a di-*ortho*-substituted congener, it is more bulky, which could be involved in its partial sequestration within cell membrane [5] and its slower mobilisation from adipocytes. On the other hand, the dynamics of mobilisation was not influenced by the presence of the other two congeners.
